# Reversing the Approach to Null Subjects: A Perspective from Language Acquisition

**DOI:** 10.3389/fpsyg.2017.00027

**Published:** 2017-02-14

**Authors:** Maia Duguine

**Affiliations:** Linguistics and Basque Studies, University of the Basque Country UPV/EHUVitoria-Gasteiz, Spain

**Keywords:** pro-drop, null subjects, language acquisition, case, language variation

## Abstract

This paper proposes a new model for null subjects, and focuses on its implications for language development. The literature on pro-drop generally considers that not allowing null subjects is, informally speaking, the “default” option in natural languages, and appeals to particular morphosyntactic mechanisms in order to account for those languages in which the subject can be omitted. Shifting the perspective, the *inverse approach* postulates that pro-drop is (almost) a default grammatical setting, and that non-pro-drop results from the intervention of independent factors that block pro-drop in the derivation. The paper explores the consequences of the inverse approach in the domain of language acquisition, arguing that this model allows to account for a number of properties of child languages. It opens an avenue of research worth exploring, one that could give new solutions to old problems.

## 1. Introduction

Under specific circumstances, sentences can have subjects which, even if unpronounced, are syntactically projected (see recently Cai et al., 2014). Pro-drop, or the possibility to omit the subject of a finite construction is a phenomenon which, in theoretical linguistics, is generally studied from a comparative perspective. The central question is why certain languages allow null subjects while others do not^1^. There is thus an opposition between pro-drop languages vs. non-pro-drop languages. But these studies have very often led to an (implicitly) asymmetrical characterization of the two options. That is, in a sense, it is considered that non-pro-drop is the default option in natural languages, and that the pro-drop option has to be motivated (that is, it has to be explained by appealing to a particular grammatical mechanism)^2^. Indeed, languages are taken to need a special grammatical feature in order to allow pro-drop, such as for instance, a pronominal Agr (*cf*. Rizzi, 1982; Alexiadou and Anagnostopoulou, 1998), a [D] feature in T (*cf*. Holmberg, 2010; Roberts, 2010a), special Case-assigners (Rizzi, 1986), or uniform agreement (Jaeggli and Safir, 1989).

A further asymmetrical characterization emerges from the work on individual or groups of pro-drop languages. It is often assumed that natural languages offer multiple ways of licensing null subjects, and thus, different types of pro-drop languages are also postulated. For instance, Italian-type languages and Chinese-type languages are typically distinguished, as allowing pro-drop vs. topic-drop (*cf*. Huang, 1984), or as licensing *pro* vs. argument ellipsis (*cf*. Saito, 2007; Roberts, 2010a). Again, this implies that conceptually, we are considering non-pro-drop as being the default option, and the fact that some languages allow null subjects is taken to derive from additional features these languages have.

But we could also flip this idea, and consider that in a sense, it is null subjects being possible that constitutes the default option, and that what has to be explained is non-pro-drop, in terms of a set of cases in which subject-drop is made impossible in the derivation of the sentence. Under this view, pro-drop is the same phenomenon in Italian and Chinese; what requires an explanation is the impossibility to drop subjects in English or French, for instance.

Furthermore, viewing pro-drop as an operation that can be blocked allows us to appeal to different conditioning factors in different cases. The same way movement can be blocked by an island, or an intervener, or the landing site being already filled, there are potentially multiple ways in which pro-drop will be blocked. This has the potential to explain the variety of cases where null subjects are not allowed, across different languages (in non-pro-drop languages, but also in pro-drop languages; see below). I will call this view the *inverse approach to pro-drop* (IA).

Now, null subjects constitute one of the best-studied topics in language acquisition, which has enlightened many aspects of the discussion on the logical problem of language acquisition, the nature of variation, parametric theory, etc. (see Hyams, 2011; Hyams et al., 2015, for recent overviews of the literature). Does the IA and the shifted view it proposes have something to bring to this field? And to what extent do the developmental data, observations and generalizations that have been collected and discovered over the years conform to the model that is suggested by the IA?

Taking the IA as a reference, the goal of this paper is to open a new perspective on the topic of null subjects in the area of acquisition, and as a first step, to explore the extent to which what we know about the acquisition of the pro-drop property makes sense under the IA.

Section 2 introduces the basic components of an account of null subjects that formalizes the fundamental ideas of the IA, and briefly presents typological and empirical evidence supporting this view. Section 3 explores some of the consequences of the shift to IA for the domain of language acquisition, on whether the standard observations on the stages of acquisition of pro-drop can be accounted for straightforwardly. Section 5 gives the conclusions.

## 2. Reversing the perspective

This section introduces the basic features of the *inverse approach to pro-drop* (IA). It does not propose a full-fledged analysis of pro-drop (see Duguine, 2013, 2014 for a more elaborated proposal). Rather, it sketches a possible account that would formalize the basic ideas of the IA that were introduced above. It also discusses evidence that supports these ideas.

### 2.1. Pro-drop and non-pro-drop under the inverse approach

By characterizing pro-drop as the “default” option for a language L, we do not necessarily have to assume that pro-drop is totally free and not subject to any syntactic condition. Instead, the claim is that all natural languages satisfy the very basic syntactic condition for allowing it, and that if a language happens not to allow null subjects, this is a fact that has to be explained.

Observe the following examples from Spanish, a pro-drop language. Whereas the DP *todos los días* “all days” can alternate with a null expression in (1B), it cannot in (2B) (the null subject is represented with “[e]”):

(1)     A.  Todos los días son una fiesta.      *Spanish*
                all       the days are a    party
                Every day is a party.
           B.  No, [e] no     son una fiesta.
                 neg     neg are a     party
                 No, they are not a party.
(2)     A.  Yo salgo         de fiesta todos los días.
                 I   go.out.1sg of party every the days
                 I go out to party every day.
           B.  Yo no    salgo        de fiesta (^*^[e]).
                 I    neg go.out.1sg of party
                 I don't go out to party.

(2B) cannot be interpreted as “I don't go out to party every day.” This shows that pro-drop is subject to a syntactic constraint. But what is this constraint? It is fair to say that the basic mechanism that makes pro-drop possible is the one behind the argument-adjunct distinction: arguments can drop, adjuncts cannot. Let us assume that structural Case—in particular, nominative-assignment in the case of subjects (and, potentially, ergative)—is this mechanism (see a.o. Chomsky, 1982; Raposo, 1986; Rizzi, 1986; Jaeggli and Safir, 1989; Platzack and Holmberg, 1989, where Case is defined as the basis of the “licensing” condition for *pro*)^3^. Assuming that Case operations hold in all natural languages, this makes all languages potential pro-drop languages. In other words, by default, any language will allow null arguments. In particular, given the (arguably universal) Case-assigning properties of finite T, this analysis accounts for the availability of null subjects across languages. What has to be accounted for are thus those languages that do not allow null arguments (or more specifically, null subjects), i.e., non-pro-drop languages.

The idea, under the IA, is that in these languages, even if the Case condition is satisfied—and thus pro-drop is in principle available—, independent factors come into play which block pro-drop. This idea can be illustrated with cases in which null subjects are impossible in pro-drop languages. It is for instance well-known that there are no focused null subjects (*cf*. Cardinaletti and Starke, 1999). In fact, focused subjects are always overt (*cf*. Larson and Luján, 1989), as illustrated in the Spanish question-answer pair in (3) (capital letters indicate focusing):

(3)     A.  Juan ha          leído Guerra y     paz.          *Spanish*
                Juan have.3sg read war      and peace
                Juan has read War and peace.
           B.  No: lo he           leído YO/^*^[e].
                no cl have.1sg read I
                No: I read it.

That is, focus has a blocking effect on pro-drop, even in contexts in which the subject satisfies the conditions for being null (i.e., it is assigned nominative Case).

In line with on this observation, the hypothesis I will put forth under the perspective of the IA is that non-pro-drop languages are languages in which there is always, in the derivation, something that blocks pro-drop. What could it be? There is a long-standing hypothesis in the literature on pro-drop, that connects the “richness” of subject-verb agreement morphology with the availability of null subjects. Indeed, pro-drop languages that have agreement morphology—such as Spanish or Italian—tend to have “rich” inflectional systems (with different forms for different person-number affixes), whereas non-pro-drop languages such as English or German tend to have many syncretic forms, i.e., “poor” agreement. This is the so-called “Taraldsen's generalization” (Taraldsen, 1980; Jaeggli and Safir, 1989)^4^. Many analyses have built on this generalization, defending that “rich” agreement is what makes null subjects possible (*cf*. Barbosa, 1995; Alexiadou and Anagnostopoulou, 1998; Speas, 2006). Following the logic of the IA, I would like to suggest here that we reverse the perspective, and postulate that in fact, “rich” agreement is not a condition on pro-drop; instead, it is “poor” agreement that blocks pro-drop.

This hypothesis can be formalized using Frampton's (2002) and Müller's (2006, 2008) characterization of poor inflection as impoverished inflection. Under the Distributed Morphology approach, impoverishment is an operation that deletes morphosyntactic features on abstract morphemes in certain specific contexts (*cf*. a.o. Bonet, 1991; Halle, 1997; Harley and Noyer, 1999). A morpheme which undergoes this operation ends up with a set of features less specified than it was before the operation took place. Frampton (2002) and Müller (2006, 2008) propose that in languages such as German, there are certain impoverishment operations which systematically delete (valued) φ-features on T, leading to the feature-specification of different morphemes being identical and thus to have the same phonological realization.

Crucially, developing the intuition that poor agreement is actually impoverished agreement, Müller (2006) makes the following suggestion: impoverishment actually bleeds pro-drop. He explains non-pro-drop in the following terms^5^:

(4)     *Pro* generalization (Müller, 2006)
          An argumental *pro* DP cannot undergo Agree with a functional head α if α has been subjected (perhaps vacuously) to φ-feature neutralizing impoverishment in the numeration.

That is, like any subject DP, *pro* enters a φ-Agree relation with T. But in contrast to DPs, it cannot enter such a relation if T has been impoverished. This would account for why subjects are necessarily overt in languages in which φ-features on T undergo impoverishment (see also Roberts, 2010b; Duguine, 2013).

Summarizing, the IA postulates that pro-drop “comes for free” in natural languages, and that non-pro-drop is what must be accounted for. As a way of formalizing this idea, on the one hand, I have proposed that structural Case-assignment is what makes null arguments available. Under the assumption that Case relations are a pervasive feature of languages, this implies that all languages are, in principle, potential pro-drop languages. It also accounts for pro-drop in all types of languages in which arguments can be null. In particular, it invites to a unified analysis of Italian-like and Japanese-like pro-drop languages (see Duguine, 2014 for arguments in favor of this unification). On the other hand, in order to account for languages that do not allow null subjects, I have appealed to the analysis proposed by Müller (2006, 2008), whereby non-pro-drop results from independent factors: impoverished T cannot combine with a null subject.

Note finally that the explanation of the non-pro-drop option in terms of impoverishment is just one example of how pro-drop can be blocked. The case of focus, discussed above, shows that there can in principle be many different ways in which different factors affect pro-drop. For instance, it has been proposed that the fact that English is not a null subject language results from T requiring an overt specifier (cf. Holmberg, 2010). If this analysis is on the right track, then it could be that in this case it is not impoverished inflection that blocks pro-drop, but rather this overtness condition on Spec,TP. The IA thus leads to a potentially multimodular and multifactorial characterization of the (non-) pro-drop phenomenon.

### 2.2. Typological and empirical evidence

The picture offered by the IA is rather unusual: it implies that pro-drop is a universal phenomenon, available in principle across all languages, with exceptions that will have to be accounted for on independent grounds. Nonetheless, as expected under this view, the availability of null arguments seems to be the unmarked option cross-linguistically.

Null arguments are licensed in the majority of the languages of the world. The broadest survey of pro-drop is probably the one by Dryer (2013), in the *World Atlas of Linguistics Structures*, which focuses on the way in which subjects are—or can be—expressed. Spanish-type languages and Japanese-type languages (i.e., pro-drop languages with and without agreement) represent 70% of the sample of languages analyzed by Dryer (2013) (498 out of 711). On the other hand, languages in which “pronominal subjects are expressed by pronouns in subject position that are normally if not obligatorily present” (English, German, French, Icelandic, etc.) represent 11.5% of the total number of languages^6^. 70% constitutes a very large majority, and the quantitative difference between pro-drop languages and non-pro-drop languages is significant^7^.

Also, the IA characterizes non-pro-drop as a property of derivations, and not as a defining property of languages. Precisely, there is a sense in which non-pro-drop languages are not fully non-pro-drop, given that there are cases, contexts or varieties in which they allow null subjects. For instance, (i) subjects of imperatives tend to be null (*cf*. Bennis, 2006 on Dutch), (ii) null subjects of finite matrix and embedded clauses are observed in certain varieties of English, such as diary British English (Haegeman and Ihsane, 2001) or Colloquial Singapore English (Sato, 2011; Sato and Kim, 2012), (iii) null subjects are also licensed in certain varieties of French—one of the few non-pro-drop Romance languages (*cf*. Roberge, 1990; Zribi-Hertz, 1994, as well as Roberts, 2010b for a critical review of the data), and (iv) Rosenkvist (2009) emphasizes that, even if null subjects are licensed in none of the modern Germanic standard languages, they are in many modern vernaculars (Zürich German, Schwabian, Bavarian, Lower Bavarian, Frisian, Övdalian and Yiddish).

In sum, the dichotomy between “pro-drop languages” vs. “non-pro-drop languages” has been largely overestimated in the literature. Indeed, the cross-linguistic data suggest that allowing null subjects is the default option for languages, and that we are not dealing with a phenomenon deeply rooted in the nature of languages, but rather the result of the conspiracy of unrelated factors affecting the derivation, as implied by the IA.

## 3. A new perspective on pro-drop in acquisition

Given the approach outlined in the previous section, the obvious question from a developmental perspective is to ask whether it can help us reach an explanation of the acquisition process. Indeed, the IA's shift regarding the question of null arguments does not have consequences for the theory of syntax only; it also affects how acquisition of the pro-drop property is expected to take place. This section explores the question of whether the IA makes sense from the point of view of language development. To that end, it briefly reviews a set of basic facts that have been established in the literature on the acquisition of (non-)pro-drop, and attempts to evaluate whether they correspond to what we could expect under the IA.

### 3.1. Early subject omission in pro-drop languages

Speakers of pro-drop languages show target-like behavior from very early on (see Valian, 1990; Guasti, 1993/1994 on Italian, Valian and Eisenberg, 1996 on European Portuguese, Wang et al., 1992 on Chinese, Kim, 1997 on Korean among others).

Under the IA, pro-drop is a default or given property of languages^8^. Therefore, the observation that children acquiring a pro-drop language show a target-like behavior is consistent with what we could expect given the IA. It is nonetheless important to note that this is not a prediction. The syntax of pro-drop is, logically, dependent on the syntax of subjects, and in particular, as proposed in Section 2, on the syntax of (structural) Case. Therefore, a child will not be expected to drop subjects until she has acquired the syntax of subjects and their Case properties (on the role of Case in the syntax and acquisition of pro-drop, see also Pierce, 1992). Consequently, what the IA predicts is that the possibility to drop the subject will follow the acquisition of the syntax of subjects. In other words, given the early acquisition of null subjects, we expect an early acquisition of the syntax of subject's Case in pro-drop languages. Precisely, acquisition of pro-drop languages seems to be characterized by an early knowledge of the syntax of subjects. For instance, in pro-drop languages, children start producing inflected verbal forms (with virtually no errors in person-agreement) and target-like subject placements very early on (*cf*. among others Guasti, 1993/1994 on Italian, Bel, 2003 on Spanish, and Barreña, 1995; Ezeizabarrena, 2002 on Basque).

### 3.2. Null subjects in early non-pro-drop languages

It is well known that early non-pro-drop languages such as English, Dutch or French allow null subjects (*cf*. Hyams, 1986). As we just saw, under the IA, given the “default” nature of pro-drop, setting the syntax of subjects is sufficient for allowing null subjects. As above, the prediction is therefore that the syntax of subjects, and in particular Case-assignment is in place from very early on in non-pro-drop languages, too.

Here, too, the prediction seems to be on the right track. Schütze and Wexler (1996) show that in early English virtually all (pronominal) subjects of finite verbs are nominative, unlike the subjects of non-finite verbs, which are often accusative (see below on root infinitives). Since in English accusative—but not nominative—is the default case (that is, DPs surface with accusative marking when they are not assigned Case; *cf*. Schütze, 2001), we can conclude with Schütze and Wexler (1996) that the fact that subjects in finite contexts are virtually always nominative shows that the syntax of nominative Case is already in place for those speakers.

### 3.3. Later setting of the non-pro-drop option

The third point is closely related to the preceding two. The observation is that whereas speakers of null subject languages seem to have a very early acquisition of the pro-drop property of their target language (i.e., what Hoekstra and Hyams, 1998 call “early morphosyntactic convergence”), the speakers of non-pro-drop languages seem to set it later (Valian, 1990). That is, they stop omitting subjects at a later stage.

Again, the IA as formalized in Section 2 provides a natural framework for these facts. Non-pro-drop requires the child to acquire the particular grammatical property or rule that blocks pro-drop^9^. What type of evidence leads to positing blocking rules? If the morphosyntactic analysis in Section 2 is on the right track, then impoverishment rules can have this blocking effect. In this case, children would posit them on the basis of evidence from inflectional morphology: there are regularities in the syncretisms across inflectional paradigms which signal rules of impoverishment. We could further conjecture that the assumption that the regularities in verbal paradigms are rule-based and not accidental is reinforced by the observation that as a derivational side-effect, these rules block subject-drop. If children are aware that adult language produces overt subjects where their own grammar (and their discourse-pragmatic knowledge) would allow them to drop subjects (see Section 3.4), positing rules of impoverishment allows them to reach a more target-like production. In other words: impoverishment rules explain two apparently independent properties of adult language. Then, if we were to explain the syntax of English overt subjects on the basis of the overtness condition on Spec, TP that we alluded to in Section 2 (*cf*. Holmberg, 2010), we would have to appeal for instance to the possibility of *indirect negative evidence* playing a role in acquisition (*cf*. Chomsky, 1981) and propose that the fact that subjects—and in particular non-referential expressions such as expletives—are systematically overt in adult production supports the assumption that there is a requirement on Spec,TP being overt.

Now, in our analysis, these rules are contingent on the syntax of subjects, and therefore it is to be expected that they will be acquired later than the property making pro-drop possible^10^. Let us take for instance Müller (2006) explanation of the non-pro-drop property in terms of morphological impoverishment. This instance of impoverishment affects the φ-features on T. These features, in turn, result from φ-Agree between T and the subject (*cf*. Chomsky, 2000, 2001). This means that φ-Agree has to be in place by the time the child learns what the rules of impoverishment of her target language are. Given the implicational relation between Case and Agree (Chomsky, 2000, 2001), we can say that the syntax of subjects, as a whole, precedes the acquisition of the rules of impoverishment. The same dependence with respect to Case and Agree occurs with Holmberg's (2010) analysis in terms of the overtness requirement on Spec,TP. In order to determine that Spec,TP must be overt, it is necessary to know that it is the subject that is realized there, and that it moves to that position because it Agrees with T. Consequently, with both possible explanations of the non-pro-drop property that we considered in Section 2, it is expected that children will go through a stage in which null subjects are allowed before showing a target-like behavior, where subjects will necessarily be overt. All in all, then, the IA provides a straightforward explanation of what was a rather mysterious consequence of earlier parametric analyses, whereby for instance Italian-speaking children seem to set the parameter relatively earlier than English speakers (see Section 4).

Finally, the impoverishment-based analysis makes a further prediction. Speakers of non-pro-drop languages are expected to take longer than speakers of rich agreement languages before they master verbal inflection. Indeed, acquisition studies show that the production of verbal inflection in early pro-drop languages is virtually errorless and displays higher rates than in early non-pro-drop languages (cf. Hyams, 1991; Phillips, 1996). However, this does not necessarily imply that in the later the inflectional system is not in place: the absence of verbal inflection corresponds in general to the use of root infinitives, and inflected forms, when produced, are also used correctly, which suggests that independent factors could be at play here (cf. Poeppel and Wexler, 1993; Phillips, 1996). More research is thus needed before we can draw conclusions on this issue.

### 3.4. Frequency

The IA characterizes pro-drop as the “default” option. One could think that this directly predicts that the frequency and distribution of null subjects in all early languages should be very similar to that of adult pro-drop languages. However, the IA does not actually make such a prediction. Indeed, pro-drop does not solely depend on structural conditions such as the Case condition discussed above. Completely independent factors also affect the distribution of null vs. overt subjects in the discourse in adult pro-drop languages. For instance, information structure (as mentioned above regarding focus in example 3) and discourse-related factors such as the accessibility or salience of the antecedent play a crucial role in deciding whether and in what context an argument can be null (Grimshaw and Samek-Lodovici, 1998; Frascarelli, 2007)^11^. Therefore, the process of acquisition of (non-)pro-drop can also only be understood by combining the grammatical level with the discursive-pragmatic level (*cf*. Hyams and Wexler, 1993 for discussion).

But to what extent do children adhere to discourse conditions on argument omission? Serratrice (2005) shows that like adults, Italian-speaking children tend to realize overtly the arguments that are discursively informative (i.e., those that do not have a salient and accessible antecedent), and to drop those that are uninformative from an early age. Other researchers, such as Clancy (1997) and Allen (2000) obtain comparable results with early Korean and early Inuktitut, respectively.

So, both the syntax of Case and the discourse-pragmatic conditions are acquired early. Therefore, the IA predicts that the frequency and distribution of null subjects in all early pro-drop languages should be very similar to that of adult pro-drop languages. And this is indeed confirmed in languages such as Italian (*cf*. Valian, 1990; Lorusso et al., 2005; Serratrice, 2005), Spanish (Bel, 2003), and Catalan (Cabré Sans and Gavarró, 2006)^12^.

But what about non-pro-drop languages? Does the IA predict that the frequency of null subjects will be the same as in adult pro-drop languages, too? Again, even if pro-drop is syntactically licensed in child languages (due to the early acquisition of the syntax of Case), frequency is also expected to depend on other factors, and in particular on the discourse-pragmatic conditions discussed above. In their study of early English, Hughes and Allen (2006, 2013) report that the more accessible the referent of a subject is, the more likely it is to be null, and the less accessible it is, the more likely it is to be overt, just like in pro-drop languages (see also Guerriero et al., 2001 on later stages of acquisition)^13^.

However, it is well known that the rates of subject-drop in early non-pro-drop languages are much lower than in pro-drop languages. According to Valian (1991), English-speaking children drop subjects at a much lower rate than Italian-speaking children (30% vs. 70%), and Wang et al. (1992) found that the 2-year old English-speaking children in their study showed far fewer null subjects than the Chinese-speaking children (approximately 26% vs. 53%). Under the IA model, null subjects are grammatical in early English. Therefore, the quantitative difference must be explained on independent grounds. What I would like to suggest is the following. English-speaking children, even though they have not yet figured out the grammatical property behind it, are aware of the low frequency (or absence) of null subjects in the adults' grammar. Thus, they produce less null subjects than what the grammar allows (see also Hyams, 1994; O'Grady, 1997 for similar ideas). This is in accordance with the findings in Hughes and Allen (2006, 2013), whereby even though the most highly accessible referents are not always null, they are much more likely to be null than the ones that are less accessible. That is, the discourse-pragmatic factors are comparable to those of Italian, and the patterns are similar, except that overall, the pro-drop option will be appealed to less often.

The difference in the frequency of null subjects between early English and, say, early Chinese or Italian is not something that should surprise us. Variation among adult pro-drop languages is also observed cross-linguistically. For instance Toribio (2000) reports that Dominican Spanish has lower rates of null subjects than Peninsular Spanish, Posio (2012) shows differences between Peninsular Spanish vs. European Portuguese, and Russian can also be taken to be a pro-drop language that omits subjects at very low rates (McShane, 2005)^14^.

### 3.5. Grammatical properties of early pro-drop

Besides the timing of acquisition and issues such as the frequency of null subjects, any adequate approach to the early stages of the acquisition of pro-drop should be able to explain the grammatical properties of null subjects in early grammars. Some observations have been made in this regard in the literature, concerning in particular the null subjects produced in early non-pro-drop languages. Some of them are discussed here, arguing that the IA provides a promising framework for their analysis.

#### Expletives

Valian (1991) and Wang et al. (1992) observe that, together with null expletives and null referential subjects, English-speaking children produce overt expletives.

This is expected under the explanation given in Section 3.4 of the higher frequency of overt subjects in early non-pro-drop languages as compared to early pro-drop languages. These children, we have seen, have a pro-drop grammar, which of course allows null expletives. But as a way to converge more closely with the adult's production, where factually, expletives are always overt, they produce less null expletives than what their grammar allows. Note that the alternation between overt and null expletives is not an issue for the claim that early English has a pro-drop grammar, since such patterns are observed in certain adult languages, such as Dominican Spanish (*cf*. Toribio, 2000) and Finnish (*cf*. Holmberg, 2005), which display overt expletives together with null expletives.

#### Root infinitives

In non-pro-drop languages, null subjects are found mostly in non-finite contexts (*cf*. the overview in Hyams, 2011). How can the IA account for them?

In adult grammars, nonfinite structures can host another type of null subject, standardly referred to as PRO (*cf*. Landau, 2013 for an overview). The first issue is therefore to determine whether the nonfinite null subjects in child grammars are of the *pro*-type or not. Now, in the analysis sketched in Section 2, Case was defined as the condition on pro-drop. Therefore, if we can determine whether in these structures there is a T that assigns Case to its subject, we will be able to characterize the nature of the null subjects they host.

In the early stages of acquisition of non-pro-drop languages, children produce target-deviant constructions with non-finite verbs in root contexts: the so-called root infinitives (or optional infinitives; see Wexler, 2011 for an overview of the literature). Schütze and Wexler (1996) showed that in English-speaking children's root infinitive structures, about half of the times the (pronominal) subject, if overt, is realized with default accusative case (while in finite contexts the subject is almost always nominative; see Section 3.2). They take this to indicate absence of Case-assignment to the subject (data from Wexler, 2011, p. 66):

(5)     a.  Him fall down. (Nina, 2;3.14, File 17)
           b.  Her have a big mouth. (Nina, 2;2.6, File 13)

Root infinitives are among those nonfinite structures where subjects are omitted. Therefore, given that no nominative Case-assignment takes place here, this subject omission does not fall under the analysis put forth here, and will have to be accounted for independently. In fact, it has indeed been proposed that these null subjects are another type of object, possibly PROs (*cf*. Sano and Hyams, 1994; Bromberg and Wexler, 1995; Schütze and Wexler, 1996; Wexler, 1998)^15^.

#### More finite-nonfinite asymmetries

There are some finite contexts in which null subjects are impossible in early non-pro-drop languages. Null subjects are very infrequent with modals (which are inherently finite in English), with finite forms of the copula such as *is, am, are*, in subordinate clauses or in finite wh-questions (e.g., *Where [e]/he/him going?* vs. ^*^Where [e]/he goes?) (*cf*. Roeper and Weissenborn, 1990; Valian, 1991; Sano and Hyams, 1994; Bromberg and Wexler, 1995; Roeper and Rohrbacher, 2000). Given the finite nature of the verbs, these cannot be contexts in which the subject is not assigned Case; therefore the explanation will have to be framed in terms of pro-drop being blocked, i.e., there being independent factors that render subject omission impossible. Have children in early stages already learned specifically that agreement on modals and copulas undergoes impoverishment (or that in those constructions SpecTP must be overt, if we adopt Holmberg's, 2010 analysis)? This is highly speculative, but it converges with the observation that even in early pro-drop languages the frequency of subject omission varies with verb class (*cf*. Guerriero et al., 2001; Allen and Schroeder, 2003; Lorusso et al., 2005). Alternatively, are they postulating another blocking constraint? In this case, what could it be? The non-finiteness restriction on post-wh null subjects, and the impossibility for null subjects in embedded contexts are even more striking: is there something in these CP areas that can block pro-drop?

This is still a poorly understood set of phenomena, and more research is needed before we can make any serious attempt for an explanation. I believe nonetheless that the IA can offer a novel and interesting viewpoint for approaching them. In fact, given that it explains non-pro-drop on the basis of the blocking of pro-drop, it predicts that there may be construction-specific properties in these finite constructions that make subject-drop impossible^16^.

## 4. Parameter (mis-)setting and the inverse approach

Hyams (1986) developed a grammar-based approach to the acquisition of (non-)pro-drop which provided support for the Principles and Parameters framework (Chomsky, 1981), arguing that early subject omission in English children's speech was due to the “missetting” of the null subject parameter (more precisely: the *AG/PRO parameter*). The idea is the following. Language acquisition consists in identifying the values of the target language's parameters. Nonetheless, these have a default setting, and the child will change the value of the parameter only if this setting does not account for the input data. In the case of null subjects, Hyams argues, the parameter's default value is positive, which is the value it has in adult languages such as Italian. This explains why early grammars of languages such as English allow pro-drop in a similar way that Italian does.

In following work, Hyams explores the hypothesis that the pro-drop phenomenon is (in part) the by-product of inflectional phenomena, and that null subjects are licensed in early grammar because of the (mis-)setting of a parameterized property of inflection (Jaeggli and Hyams, 1988; Hyams, 1991). More precisely, she adopts Jaeggli and Safir's (1989) analysis of null arguments, whereby null subjects are licensed only in languages with uniformly inflected or uniformly uninflected verbal paradigms, that is, with paradigms composed of complex forms only—i.e., different forms for all person-number combinations, as in Italian—, or with no complex form whatsoever, as in Chinese (the *morphological uniformity principle*)^17^.

Jaeggli and Hyams (1988) and Hyams (1991) propose that null subjects are allowed in early English because children's initial assumption is that the language's morphological paradigm is uniform. Thus, shifting to a non-pro-drop grammar requires them to “realize” that the verbal paradigms are not uniformly inflected.

The analysis of the pro-drop phenomenon proposed in Section 2 shares important aspects with some hypotheses adopted in the parameter (mis-)setting approach; in particular, the idea that the pro-drop phenomenon is (at least in part) the by-product of the properties of inflection. Leaving the theoretical aspects aside (for discussion see Duguine, 2013: chapter 6), what follows discusses the similarities that concern the issue of acquisition. Indeed, in both analyses, early grammars (i) allow pro-drop and (ii) have “uniform” verbal inflectional morphology. Logically then, many predictions made by the IA are also made by Hyams' proposal: null subjects in early pro-drop and non-pro-drop languages, later setting of the non-pro-drop option, dependency of the setting of the non-pro-drop option on the acquisition of the properties of inflection, etc^18^.

Different aspects of Hyams' accounts reported above have been challenged conceptually and empirically. What follows discusses three problems that concern basic aspects of Hyams' proposal and shows that adopting the perspective of the IA offers a way to avoid them.

Hyams' parametric approach faces three important issues (see Hyams, 2011 and references therein). First, it does not conform to the Subset Principle, whereby children posit the parameter value that generates the most restricted language consistent with their input data. Indeed, since the positive value of the pro-drop parameter in Italian allows both overt and null subjects, it is a superset of the negative value of English, which only allows overt subjects. Therefore, the Italian setting could not be the initial one. Second, an issue arises with respect to the timing of parameter setting. Data indicates an early setting of the parameter in pro-drop languages, while children acquiring non-pro-drop languages still produce null subjects (see Section 3.3). But if the parameter is set early in Italian, it should also be set early in English. And the third problem is raised by how the explanation based on the morphological uniformity principle is applied to the case of English-like languages. If early English has uniformly uninflected verbal paradigms (just like Chinese), then children beginning to produce inflectional morphology indicates that they have reset their grammar as having non-uniform verbal paradigms. The prediction is thus that they will simultaneously exit the null subject stage. However, this is not borne out: children produce null subjects even after they begin using inflectional morphology.

All three of these issues can be linked to a particular feature of Hyams' analysis: it relies on the existence of a dedicated parameter for pro-drop. As is standardly accepted under the Principles and Parameters framework (*cf*. Chomsky, 1981 and ff.), cross-linguistic variation in the availability of pro-drop in the syntax depends on the predetermined set of values of a dedicated parameter. But what if the pro-drop phenomenon was not the (direct) product of a parameter? What if there was no *Null Subject* (or *AGR*, or *morphological uniformity*) *Parameter*?

This is precisely a hypothesis that can be considered and explored under the IA. Indeed, (even) within the Principles and Parameters framework, the model that emerges from the analysis sketched in Section 2 does not conform to that of a parameter, since it postulates that variation in pro-drop emerges from the interplay between different components of the grammar (for discussion see also Duguine et al., in press). It could be considered that the syntax of Case, the rules of impoverishment, and/or the requirement on an overt Spec,TP are parameterized properties. Nonetheless, it has the following features that distinguish it from standard parametric approaches: (i) pro-drop is universally allowed, and (ii) non-pro-drop is not a core, defining property of languages; it results from pro-drop being systematically blocked in certain configurations in particular languages. This is why, under the IA variation in pro-drop will not be formally characterized as an example of parametric variation.

Crucially, this model will not face the issues that a parametric model such as Hyams' is confronted with. First, the child is complying with the Subset Principle. The IA view does not postulate that acquirers of English posit an incorrect value for a parameter. There is no parameter missetting, and there is no parameter, for that matter. Acquiring a non-pro-drop grammar requires two steps: acquiring the syntax of subjects (i.e., Case) and acquiring the blocking rule. The child makes the first step arguably on the basis of all the morphosyntactic evidence for Case that is available in her primary linguistic data (case morphology, A-movement, etc.). This property is correctly set, that is, it corresponds to the adult grammar. Since pro-drop is universal (to the extent that Case is universal) all children first posit a pro-drop grammar. But even though is true that English-speaking children's early grammar will generate a language that is a subset of their target language, this is because they have not yet acquired the properties of the grammar that prevent pro-drop. And when doing so, again, they will comply with the Subset Principle, since they will be positing the grammar that generates the most restricted language consistent with their input data.

Second, the IA also allows us to explain the delay in the acquisition of non-pro-drop grammars with respect to pro-drop grammars (see Section 3.3), and predicts that children will attain target-like production progressively, as they acquire the different components of this system, that is, the different linguistic properties that can affect (and in particular, block) pro-drop in the adult language.

Third, if the analysis in terms of impoverishment sketched in Section 2 is on the right track, the production of inflected forms is not expected to correlate with the child exiting the null subject stage. Children will ultimately have to uncover the set of rules of impoverishment affecting inflectional morphemes before they stop dropping subjects, that is, they will have to realize that the syncretisms in verbal paradigms are not accidental, that they result from morphological rules (which, incidentally, block pro-drop; see Section 3.3). That is, conceivably, until that point they can produce inflected or non-inflected forms and null subjects.

To conclude, the approach explored in this paper offers a perspective on the acquisition of pro-drop that shares important features with earlier work, in particular Hyams (1986, 1991), and makes various similar predictions. However, in contrast with these, it also implies that there is no Pro-Drop Parameter as such. The patterns of null/overt subjects across languages emerge from the conspiracy between different components of the grammar, and the stages of language development result from the timing in the acquisition of these components. This difference allows it to circumvent some problems that Hyams' proposal was confronted to. So, the IA can be seen as the opportunity to re-open the discussion on the acquisition of null arguments, and explore with new tools an account that was quite well supported both conceptually and empirically.

It must be pointed out that in the years following Hyams' work, studies showed that there are differences in the distribution of null subjects in early non-pro-drop languages vs. early pro-drop languages. These concern observations that were cited in Section 3.5: null subjects are very infrequent with modals, with finite forms of the copula such as *is, am, are*, in subordinate clauses or in finite wh-questions (e.g., *Where [e]/he/him going?* vs. ^*^Where [e]/he goes?) (*cf*. Roeper and Weissenborn, 1990; Valian, 1991; Sano and Hyams, 1994; Bromberg and Wexler, 1995; Roeper and Rohrbacher, 2000). This observation, combined with other issues such as the three points discussed above, have led many researchers to consider that early missing subjects in non-pro-drop languages are not part of the pro-drop phenomena. In particular, Rizzi (2005a,b) develops an influential account whereby null subjects in early non-pro-drop languages result from “root subject drop,” a (parameterized) grammatical option where the specifier of root/truncated clauses (bare IPs) can be null. The root subject drop analysis straightforwardly accounts for “root” effects in early English such as the impossibility for null subjects to occur after a wh-phrase or in a subordinate clause, but as noted by Hyams (2011), it does not explain why they do not occur with modals (Valian, 1991) or with finite forms of the copula (Sano and Hyams, 1994)^19^. Section 3.5 merely sketched a possible explanation for the latter facts under the IA, but I hope to have shown that, even though much remains to be done, the IA can be seen as a version of the parameter missetting approach, which circumvents some earlier problems, and which can be investigated as an alternative explanation of the acquisition of (so-called) pro-drop vs. non-pro-drop languages.

## 5. Conclusions

The *inverse approach to pro-drop* (IA) proposes a shift in the way the question of pro-drop is addressed. This paper has focused on its implications for language development, showing that it offers an explanatory account of several properties of child languages, both with pro-drop and non-pro-drop target languages. It shares important features with earlier proposals, such as in particular the parameter missetting approach developed in Hyams (1986, 1991). But there are also crucial differences. In particular, the conceptual consequence that there is no Pro-Drop Parameter as such allows us to circumvent some issues raised by these earlier accounts. In other words, the results obtained here suggest the parameter missetting approach can be brought back to the research in the acquisition of (non-)pro-drop, since they give a way to formalize the developmental intuition that all children start out with a pro-drop grammar, and that this is why those speaking a pro-drop language will show early target-like behavior and those speaking a non-pro-drop language will shift to a different grammar later on.

Much remains to be done, but I hope the above discussion succeeds in showing that the IA opens some avenues of research that are worth exploring, and which might give new solutions to old problems.

## Author contributions

The author confirms being the sole contributor of this work and approved it for publication.

## Funding

The present work was made possible through funding from the Basque Government (IT769-13), the Spanish Ministry of Economy and Competitiveness (FFI2014-53675-P, FFI2014-51675REDT), and the University of the Basque Country UPV/EHU (UFI11/14).

### Conflict of interest statement

The author declares that the research was conducted in the absence of any commercial or financial relationships that could be construed as a potential conflict of interest. The reviewer EP and the handling Editor declared their shared affiliation, and the handling Editor states that the process nevertheless met the standards of a fair and objective review.

## Footnotes

^1^For reasons of space, and in order to be able to focus on the central aims of the paper, I will leave null objects aside (see footnote 16). Therefore, in this paper I use the terms *pro-drop, subject-drop* and *null subject* interchangeably.

^2^Note that this statement is not equivalent to assuming that there is a default negative setting for the pro-drop parameter: it just aims at making explicit how researchers approach the issue of null arguments. See also footnote 18.

^3^For a discussion of evidence in favor of Structural Case as the condition on pro-drop, see Duguine (2013).

^4^“Richness” has proven to be a difficult notion to define (*cf*. attempts in a.o. Jaeggli and Safir, 1989; Rohrbacher, 1999; Müller, 2006). Nonetheless, the generalization holds, which suggests that at least at an abstract level, “richness” is relevant for pro-drop (*cf*. also Roberts, 1993; Platzack, 1994; Vainikka and Levy, 1999).

^5^Müller (2006, 2008)'s analysis relies on the assumption that Morphological Structure comes before syntax proper, so that φ-Agree—a syntactic operation—can be affected by the output of impoverishment—a morphological operation. See Duguine (2013) for an alternative analysis that maintains a standard architecture of the grammar, with a post-syntactic morphological component.

^6^The three other groups of languages—which constitute 18.4% of the sample—are not easy to classify directly as either pro-drop or non-pro-drop, but some of them, such as Warlpiri (Legate, 2006), Finnish, Hebrew (Vainikka and Levy, 1999) or Irish (*cf*. McCloskey and Hale, 1984) are known to allow null subjects. The actual proportion of null subject languages is thus larger than 70%.

^7^In the sample of 104 languages studied in Gilligan (1987), 93% are classified as null subject languages.

^8^This consequence of the IA converges with the early parameter missetting approach in Hyams (1986), Jaeggli and Hyams (1988), and Hyams (1991), which posits that pro-drop is the default option in language development; see Section 4.

^9^Above, following Haegeman and Ihsane (2001), Sato (2011), and Sato and Kim (2012) I suggested that certain varieties of English allow pro-drop. But as discussed by Mack et al. (2012) and Frazier (2015), standard English does not, and the occasional dropping of subjects results from performance factors, where predictable material is reduced. Frazier (2015) highlights that this suggests that the speakers are implicitly aware of the reduction predictable material, and that children may recognize these deviations as being due to the performance system, thus not taking them as evidence that their target is a pro-drop grammar.

^10^The proposal in Jaeggli and Hyams (1988) and Hyams (1991) similarly predicts a later setting of the non-pro-drop option as an result of children realizing late that their target language has poor agreement; see also Section 4.

^11^There are actually many other factors that influence pro-drop that we will not discuss here, such as for instance verb class (*cf*. Guerriero et al., 2001; Lorusso et al., 2005), or the understanding of the listener's mental state and perspective (Sorace et al., 2009).

^12^Some studies report higher frequency of subject omission by children than by adults, which can be explained on independent grounds (*cf*. Serratrice, 2005; Hyams, 2011). For instance, their discourse-situation is often immediate, and their interactions with adults are generally initiated by the latter.

^13^That is, children appear to overgeneralize the use of null subjects when the adult target form would be an overt pronoun or a demonstrative (Hughes and Allen, 2006).

^14^See also Camacho (2013), who proposes that in language change, the first phase of the shift from a pro-drop grammar to a non-pro-drop grammar simply involves an increase in the frequency of overt subject (without there being a change in the syntax).

^15^Rizzi (2005a,b) proposes an account that subsumes the null subject phenomenon of early non-pro-drop languages under the root infinitives phenomenon. See Section 4.

^16^An important point that has not been discussed in this paper is that of null objects. The Case condition predicts that objects—to the extent that we assume that they are assigned structural accusative Case—should be omitted during the early stages of acquisition. This is borne out, since object omission occurs both in languages with null objects and in languages without null objects (although at much lower frequencies than subject omission: about 10% in English (Valian, 1991; Wang et al., 1992, 20% in Chinese Wang et al., 1992). A discussion of objects would require to first establish assumptions on the nature of a.o. object clitics and clitic optionality in Romance languages, as well as explaining what blocks object-drop in adult languages such as English. I leave these issues open for future research.

^17^With the *morphological uniformity principle*, Jaeggli and Safir (1989) formalize Taraldsen's generalization (*cf*. Section 2), integrating in the account pro-drop languages that have no verbal agreement morphology (e.g., Japanese and Chinese).

^18^Note that even though in Hyams (1986), Jaeggli and Hyams (1988), and Hyams (1991) pro-drop is the default option in language development, it is not the default option in the syntax, in the sense considered in Section 2. Indeed, these analyses assume that the syntax of pro-drop is constrained by specific grammatical mechanisms (licensing and identification conditions), which implies that conceptually non-pro-drop is the default option.

^19^Hyams (2011, pp. 27–30) also raises more general questions (see also Serratrice and Allen, 2015). For instance, whether Italian children also posit the positive value of the parameter that makes root subject drop possible, and how the two parameters are expected to interact.
